# Data regarding dynamic performance predictions of an aeroengine

**DOI:** 10.1016/j.dib.2020.105977

**Published:** 2020-07-04

**Authors:** Maria Grazia De Giorgi, Marco Quarta

**Affiliations:** Department of Engineering for Innovation, Via per Monteroni, University of Salento, 73100 Lecce, Italy

**Keywords:** Aeroengine, Turbojet modelling, Artificial neural network, Machine learning

## Abstract

The design of aeroengine real-time control systems needs the implementation of machine learning based techniques. The lack of in-flight aeroengine performance data is a limit for the researchers interested in the development of these prediction algorithms. Dynamic aeroengine models can be used to overcome this lack.

This data article presents data regarding the performance of a turbojet that were predicted by the dynamic engine model that was built using the Gas turbine Simulation Program (GSP) software.

The data were also used to implement an Artificial Neural Network (ANN) that predicts the in-flight aeroengine performance, such as the Exhaust Gas Temperature (EGT).

The Nonlinear AutoRegressive with eXogenous inputs (NARX) neural network was used. The neural network predictions have been also given as dataset of the present article.

The data presented here are related to the article entitled “MultiGene Genetic Programming - Artificial Neural Networks approach for dynamic performance prediction of an aeroengine” [1].

Specifications table

**Table:** 

Subjectk	Engineering, Aerospace Engineering
Specific subject area	Artificial Neural Network; Aeroengine
Type of data	Table Plot
How data were acquired	The data were given by: Real in-flight data as input for GSP Nonlinear autoregressive neural network EGT predictions
Data format	Raw Analysed
Parameters for data collection	Environmental conditions (altitude, Mach number, temperature, etc.) are different for each mission and for each flight phase and are provided as input to the GSP software. The Exhaust Gas Temperature recorded in-flight is provided as a comparison and validation parameter for GSP calculations.
Description of data collection	Turbojet engine data used as input into GSP was measured by on-board sensors during eight real training missions.
Data source location	University of Salento, Dep. Engineering for Innovation, Via per Monteroni, 73,100 Lecce, Italy
Data accessibility	With the article and as supplementary files
Related research article	Maria Grazia De Giorgi, Marco Quarta, Hybrid MultiGene Genetic Programming - Artificial Neural Networks approach for dynamic performance prediction of an aeroengine, Aerospace Science and Technologies, In Press.

## Value of the data

• The data provided permit to analyse the operation of an aeroengine under transient conditions, as in-flight conditions.
• The data presented in this paper can be used by other researchers who need to apply these data to evaluate the performance of different types of machine learning techniques.
• Researchers can use these data to assess the performance of intelligent control algorithms applied to aeroengine under dynamic operating conditions.
• These data are useful for other research in order to implement on board self-tuning model for aero-engine.

## 1 Data description

The data article presents the datasets on the turbojet studied in [1] to implement the model in the Gas Turbine Simulation Program (GSP) [2].

The file “GSP Missions.xlsx" contains the data (time, altitude, ambient temperature difference from the one calculated by ISA, Mach number and engine rotational speed) from eight real flight missions used as input to validate the engine GSP model and the output exhaust gas temperature EGT predicted by the GSP software.

The file “GSP_DATA.xls” contain 8 sheets ( “GSP_Mission_1″, “GSP_Mission_2″, “GSP_Mission_3″, “GSP_Mission_4″, “GSP_Mission_5″, “GSP_Mission_6″, “GSP_Mission_7″, “GSP_Mission_8″) that report the data of the 8 flight missions. In each sheet there are 11 columns that are in order: Mach number (M), Atmospheric total temperature (*T*_t1_) [K], atmospheric total pressure (p_t1_) [bar], air mass flow rate (*W*_a_) [kg/s]; compressor pressure ratio [-]; rpm (N) [%]; turbine inlet total temperature (*T*_t4_) [K], fuel mass flow rate (W_f_) [kg/s], Exhaust gas temperature [K], Thrust [kN].

Furthermore, the results of the EGT made with NARX neural networks for the 11 input configurations specified in the related article [1] have also given in Fig. 1, Fig. 2, Fig. 3, Fig. 4, Fig. 5, Fig. 6, Fig. 7, Fig. 8, Fig. 9, Fig. 10, Fig. 11 and reported in the file “NARX_EGT.xls” that contains 12 sheets, 11 for each NARX input configuration and one for the NARX EGT target for the test of the neural network.

**Fig. 1:**
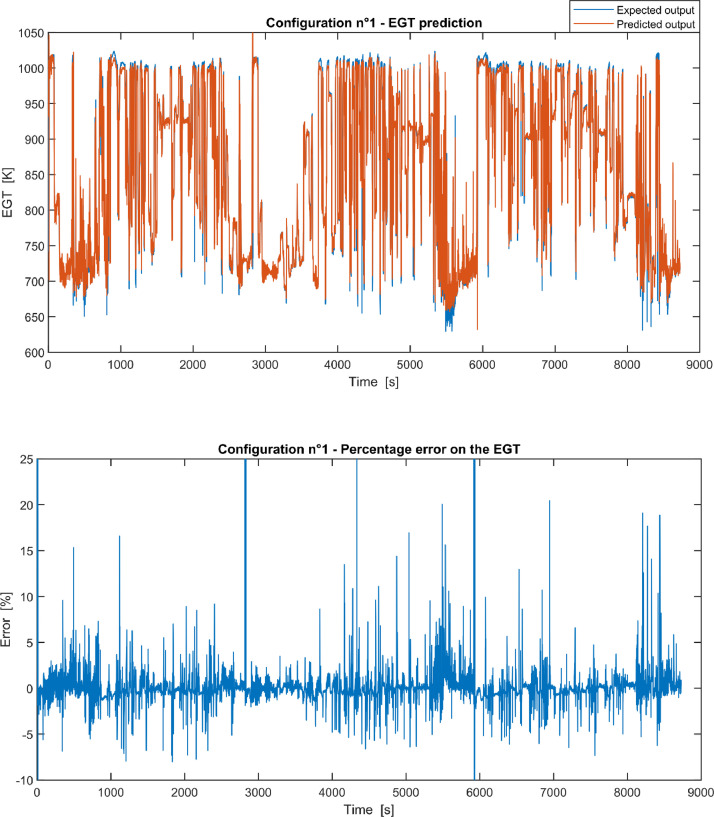
Configuration 1: Expected versus Predicted EGT – Percent Error on EGT.

**Fig. 2:**
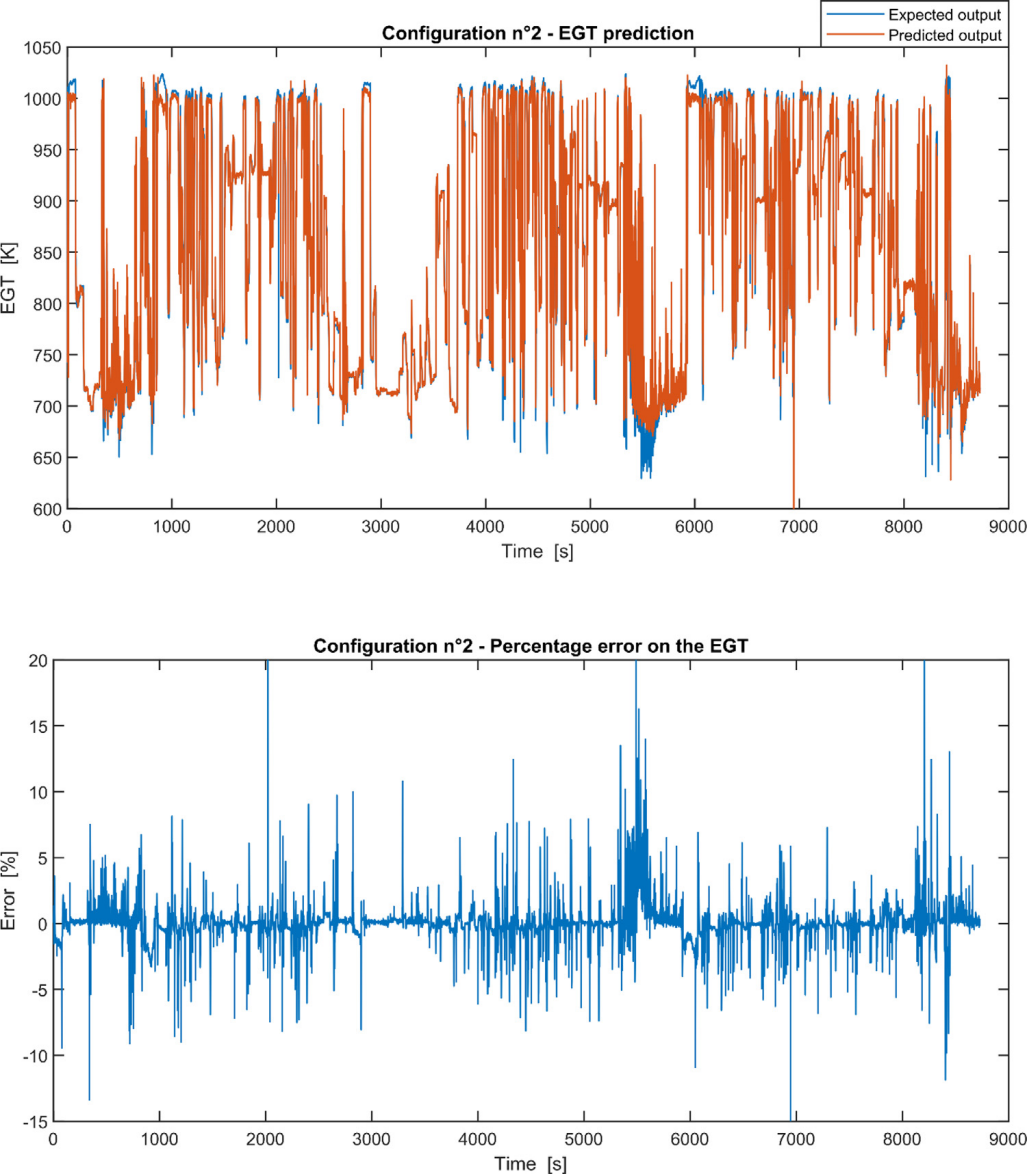
Configuration 2: Expected versus Predicted EGT – Percent Error on EGT.

**Fig. 3:**
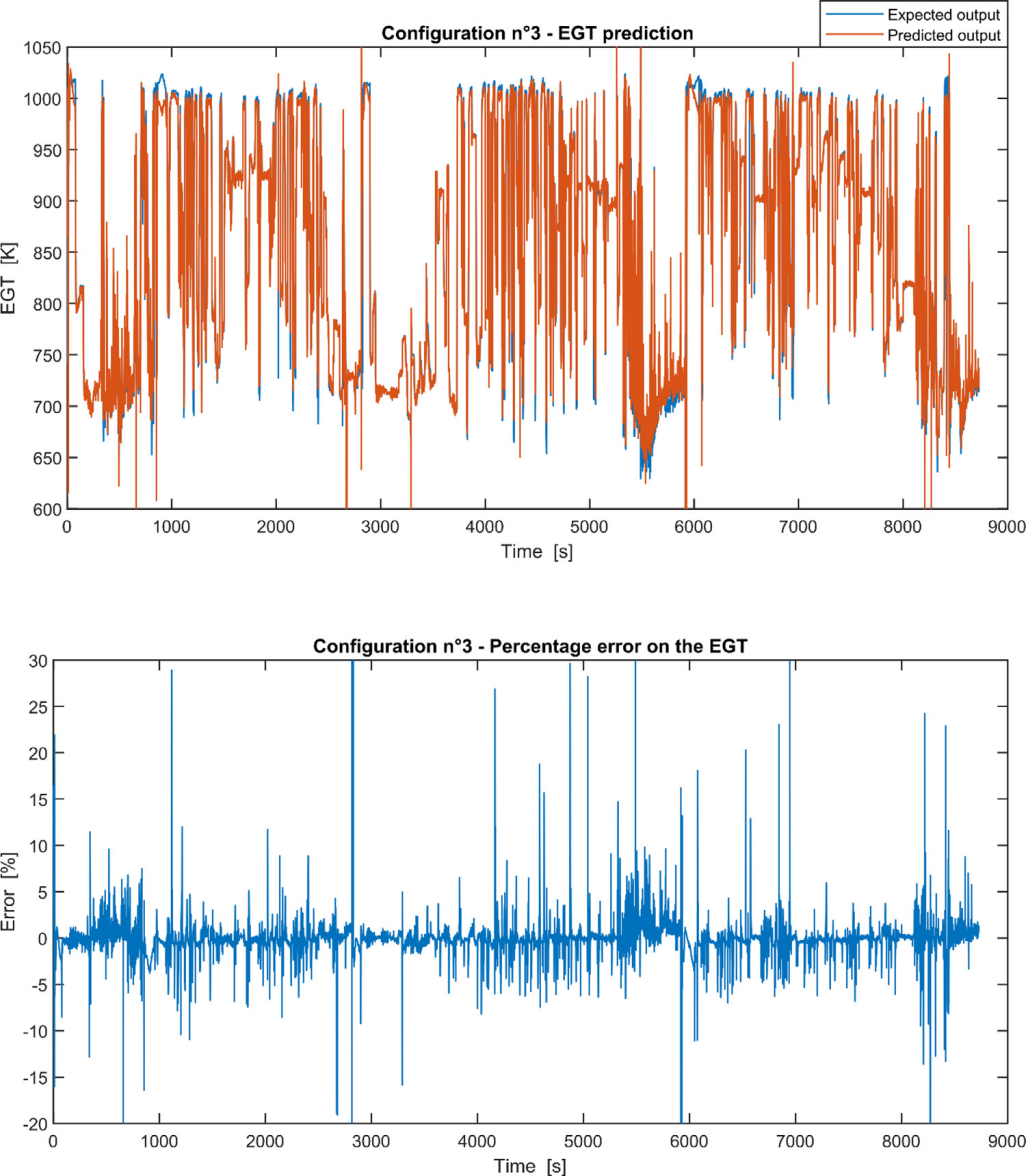
Configuration 3: Expected versus Predicted EGT – Percent Error on EGT.

**Fig. 4:**
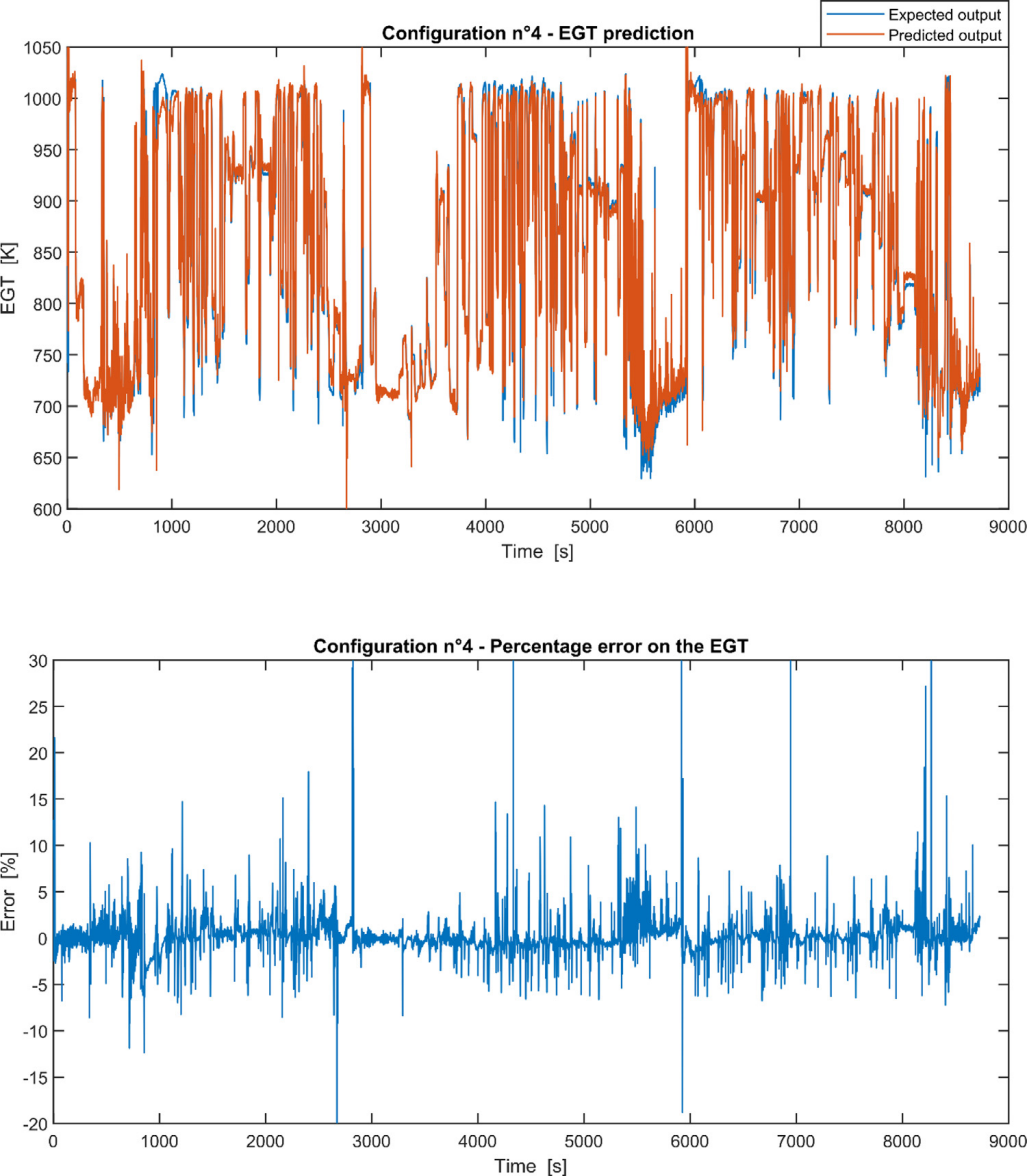
Configuration 4: Expected versus Predicted EGT – Percent Error on EGT.

**Fig. 5:**
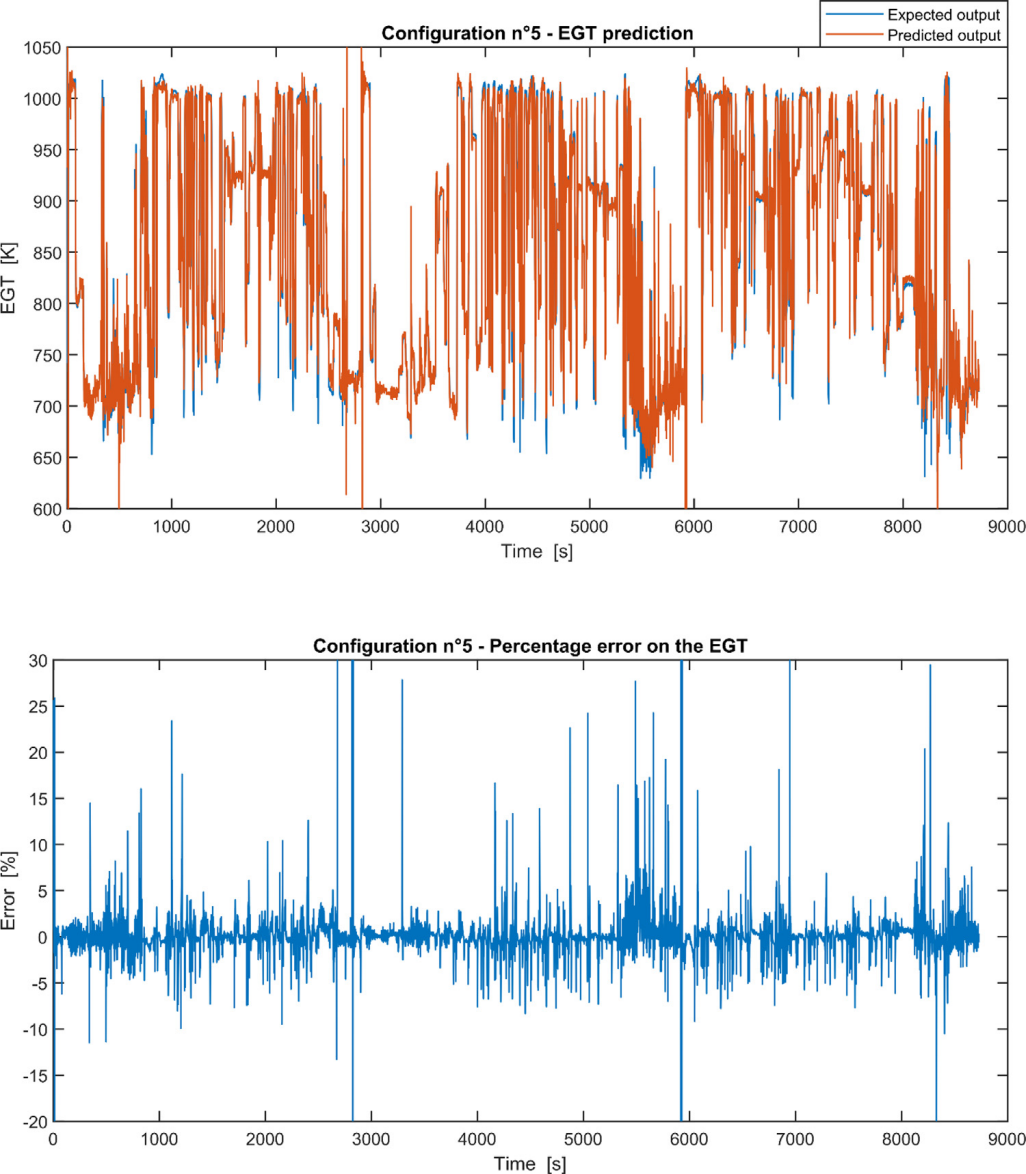
Configuration 5: Expected versus Predicted EGT – Percent Error on EGT.

**Fig. 6:**
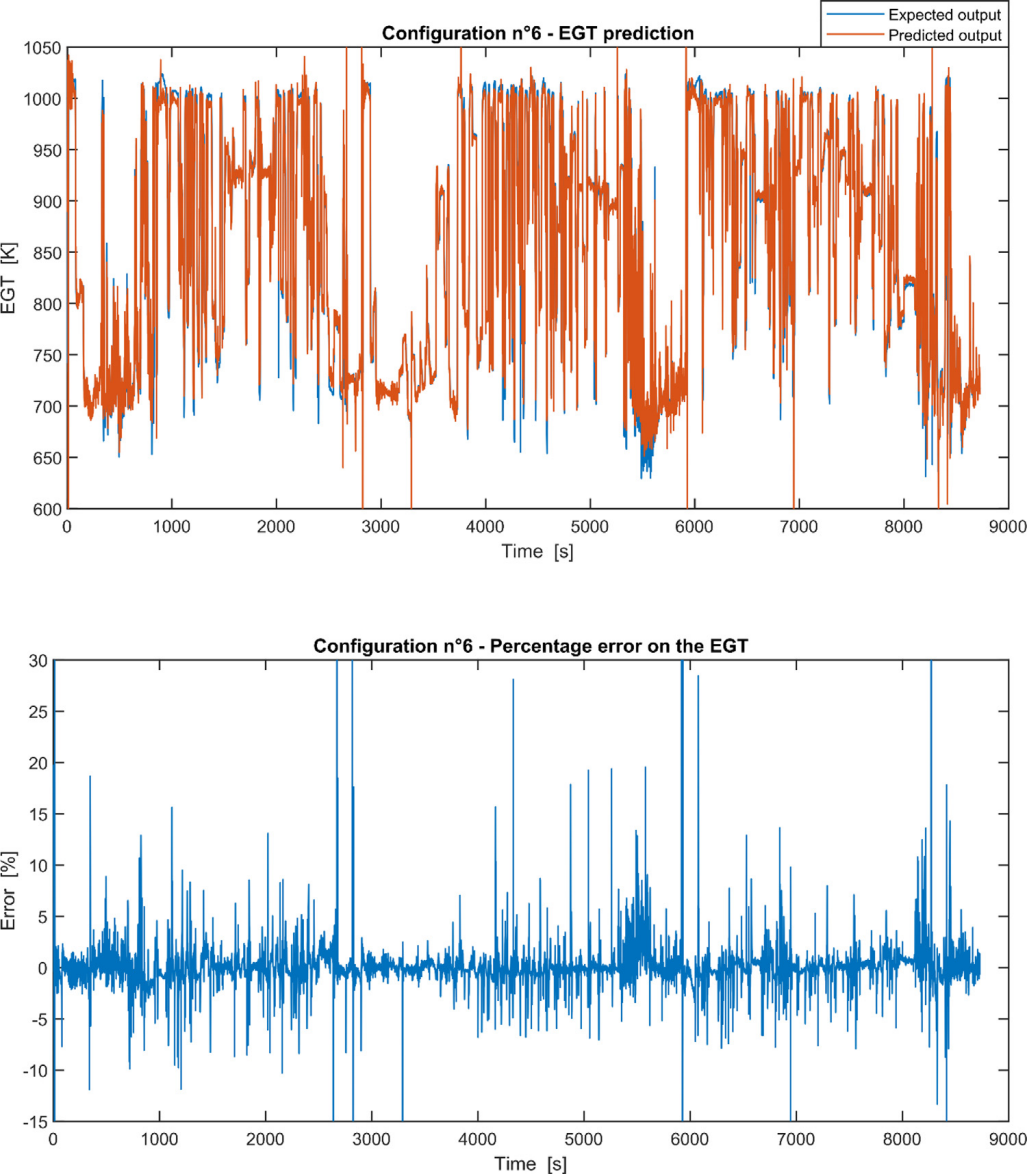
Configuration 6: Expected versus Predicted EGT – Percent Error on EGT.

**Fig. 7:**
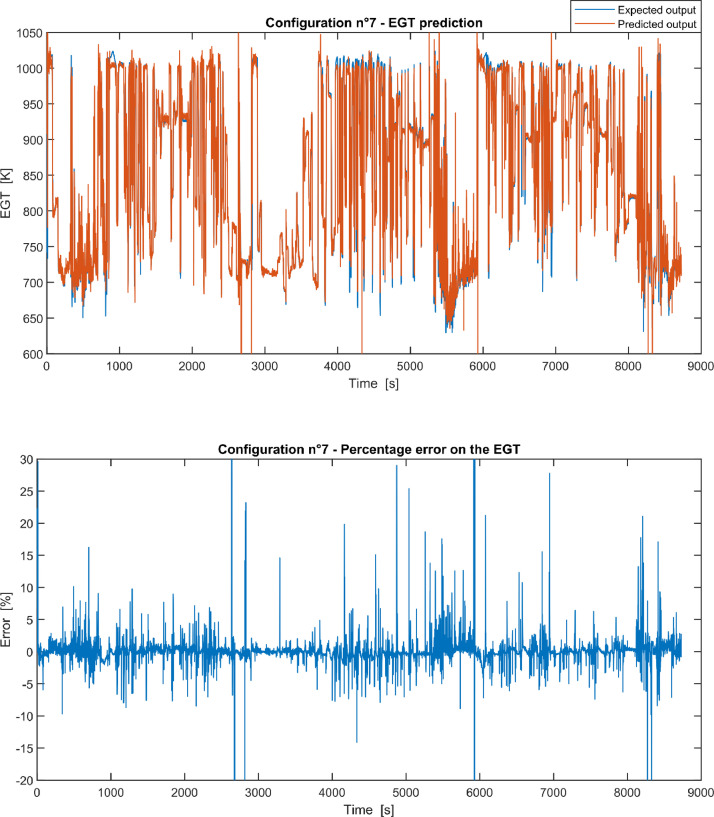
Configuration 7: Expected versus Predicted EGT – Percent Error on EGT.

**Fig. 8:**
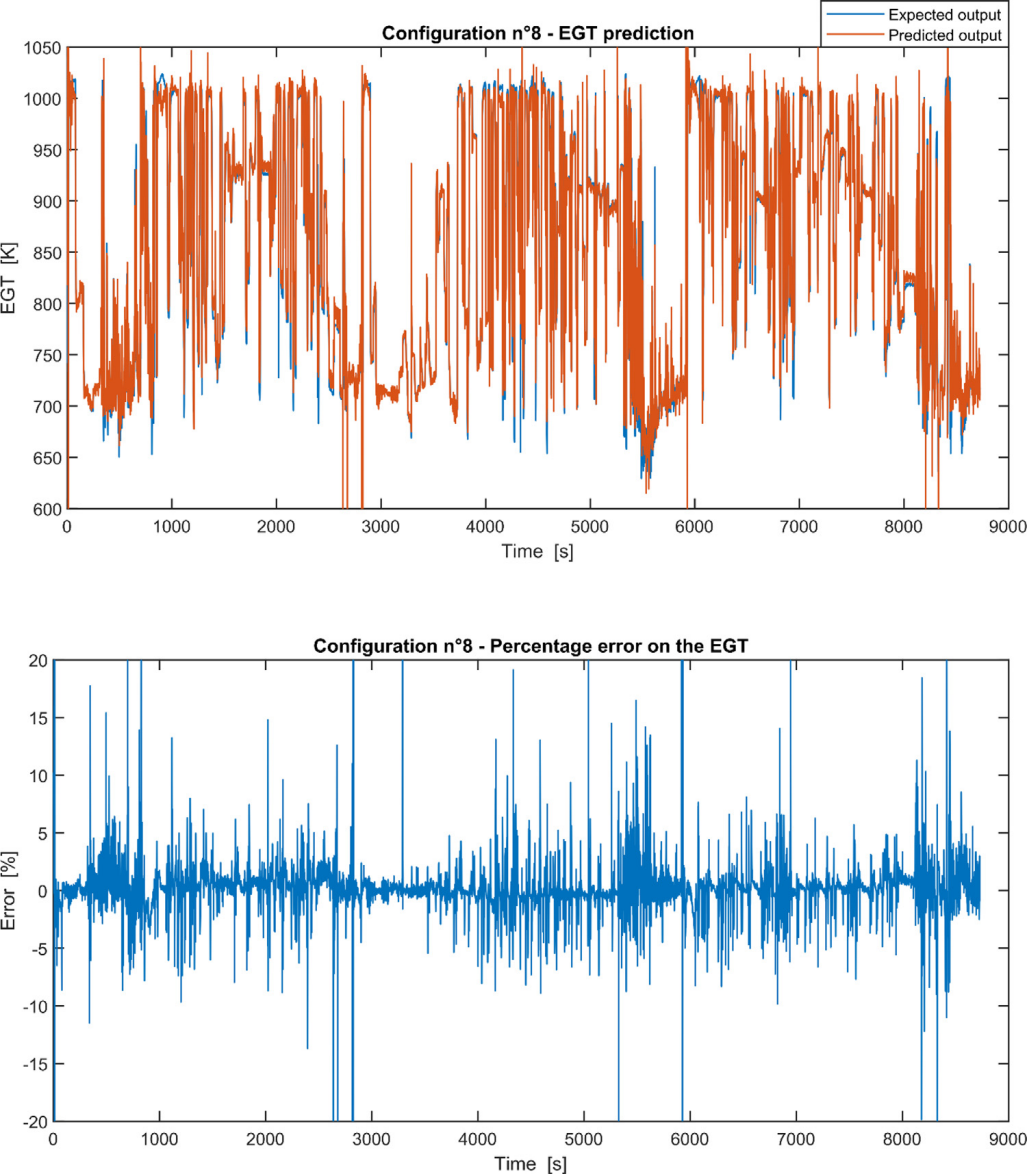
Configuration 8: Expected versus Predicted EGT – Percent Error on EGT.

**Fig. 9:**
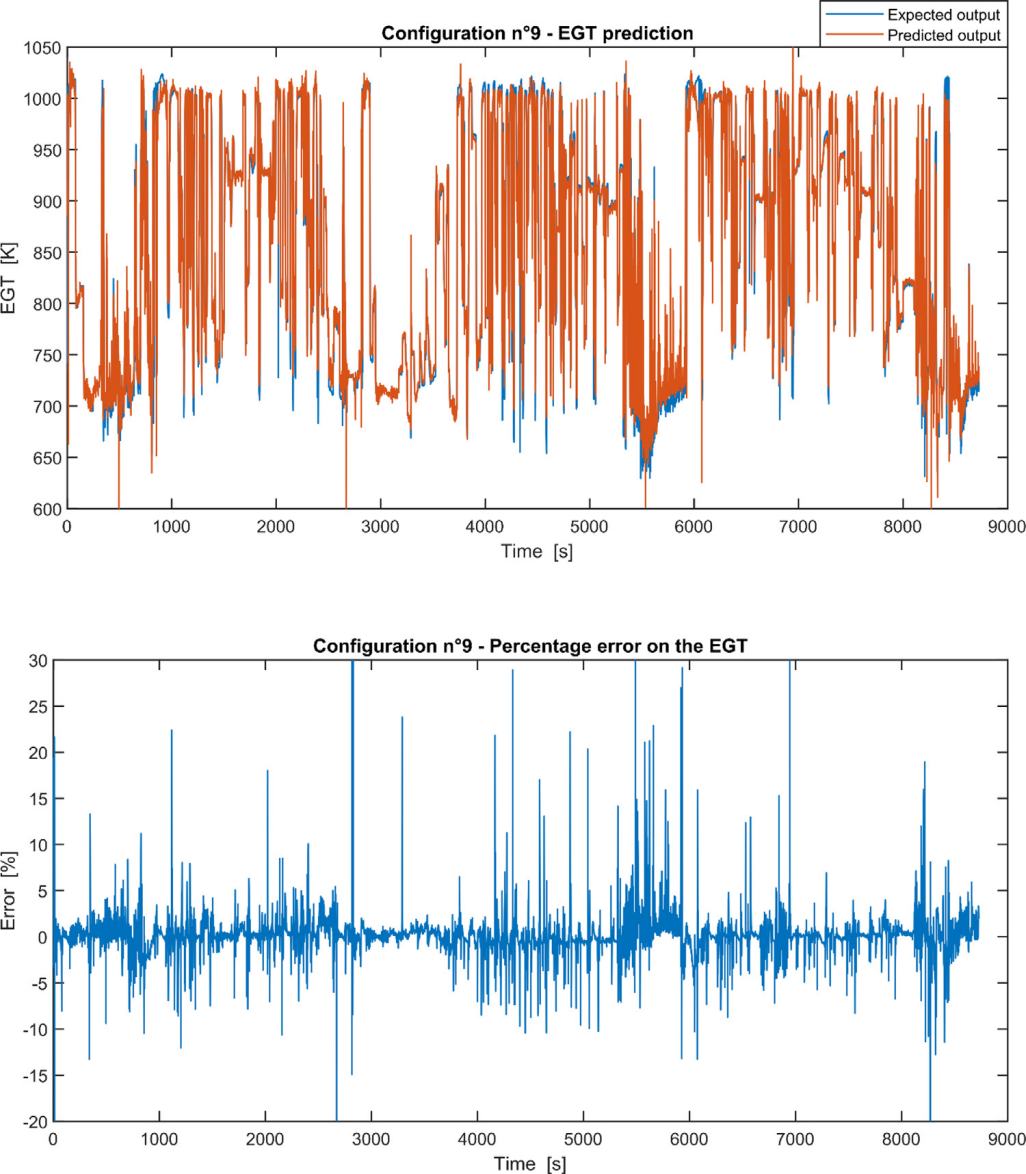
Configuration 9: Expected versus Predicted EGT – Percent Error on EGT.

**Fig. 10:**
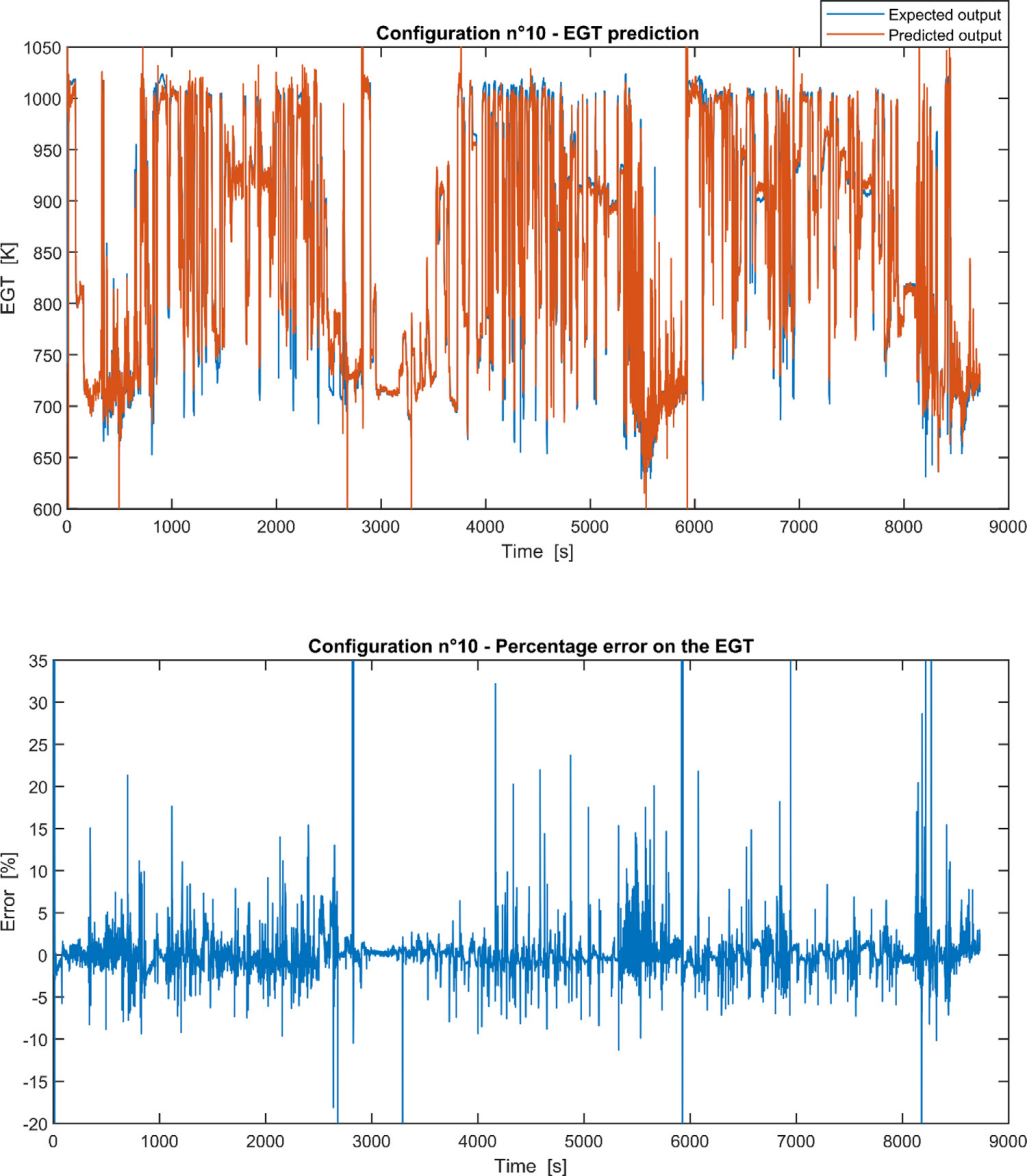
Configuration 10: Expected versus Predicted EGT – Percent Error on EGT.

**Fig. 11:**
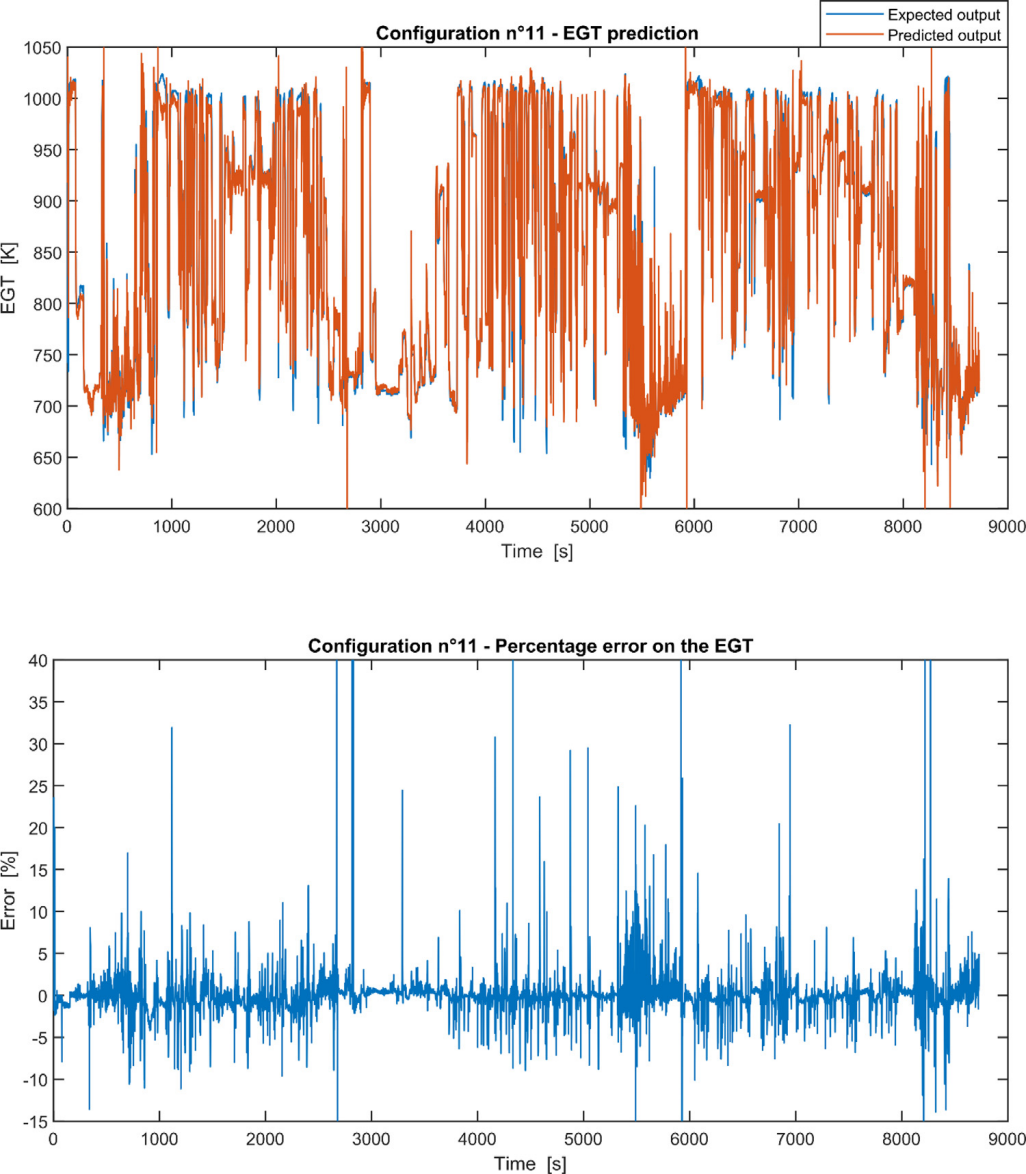
Configuration 11: Expected versus Predicted EGT – Percent Error on EGT.

## 2 Experimental design, materials and methods

The datasets regard the input and output data of the Gas Turbine Simulation Program (GSP) package [2]. In particular, the GSP software is a 0D model to predict gas turbine engine performance. Flow properties are only defined and/or predicted at each engine component inlet and exit. Components itself can be well describe by setting key parameters, such as the design compression ratio for the compressor, the mass air flow rate, the fuel flow rate, the maximum allowable temperature at the turbine inlet, polytropic and mechanical efficiencies and so on. The software can predict engine performance at design point, steady-state off-design and transient calculations. The engine description as well as the design parameters that were used in GSP to model each engine component can be found in [1,3].

Furthermore, Nonlinear AutoRegressive with eXogenous inputs (NARX) neural networks were used in [1] and the predictions on the EGT were compared with real flight data.

The NARX was implemented with six parameters given as input and output of the GSP, based on the eight missions reported in the excel file.

In particular these parameters were used: Flight Mach number (M), atmospheric total temperature (T_t1_), total pressure (p_t1_), shaft speed (N), turbine inlet total temperature (T_t4_), fuel mass flow rate (W_f_). These data are reported in the file “GSP_DATA.xls”.

In order to verify the influence of each of these six variables on the EGT, 11 different combinations of these parameters have been used as input dataset for the NARX, as illustrated in Table 1.

**Table 1:** Input configurations used for NARX (in blue the used parameters).

These 11 datasets of flight missions from #1 to #5 were used for the training of the NARX and the datasets of missions from #6 to #8 were used for the test. Each figure, from Fig. 1 to Fig. 11, contains the plot with the comparison between the real EGT, measured in flight, and the one predicted by the NARX network and the plot of the percentage error between predicted and real EGT. The NARX predicted EGT data and the real EGT are reported also in the file “NARX_EGT.xls”.

## Declaration of Competing Interest

The authors declare that they have no known competing financial interests or personal relationships which have, or could be perceived to have, influenced the work reported in this article.

## Appendix Supplementary materials

Image, application 1

Image, application 2

Image, application 3
